# Prince of Wales's Hospital Fund for London

**Published:** 1901-05-18

**Authors:** 


### THE INSTITUTIONAL WORKSHOP.

### PRINCE OF WALES'S HOSPITAL FUND FOR LONDON.

A MEETING of the General Council of the above Fund was held last week at the residence of the Duke of Fife, 15 Portman Square, his Grace occupying the Chair on behalf of H.R.H. the Duke of Cornwall and York.

There were also present Viscount Duncannon, Lord Rothschild, Lord Iveagh, Lord Farquhar, the Chairman of the School Board for London (Lord Reay), Sir Wm. MacCormac, Sir Trevor Lawrence, Sir John Aird, Sir Henry Burdett, Mr. Sydney Buxton, Mr. J. G. Craggs, Mr. F. M. Fry, Mr. H. C. Smith, Rev. T. Bowman Stephenson, D.D., and Mr. Julius Wernher.

Letters of regret were read from the Duke of Norfolk, the Bishop of London, the Bishop of Rochester, Cardinal Vaughan, the Chief Rabbi, Lord Lister, the Lord Mayor, the Chairman of the London County Council, the Governor of the Bank of England, Mr. T. Burt, M.P., Dr. Collins, Mr. Wm. Latham, K.C., and Mr. Sandeman.

The DUKE OF FIFE, in opening the proceedings, said:—My Lords and Gentlemen, I need hardly say it gives me great pleasure to hold this meeting at my house in accordance with the wish of the King. I have been asked to explain that this meeting would have been held very much earlier in the year but for the sad event which, even at this date, it is difficult to expel from one's mind, I mean the death of our late Sovereign. It is now my duty to announce the resignation of the Presidency of the Fund by the King, but I am very glad to say that His Majesty has kindly consented to become its patron, and he has directed me to say that he will always continue to take a deep interest in the work which it performs. I have just received the following telegram from the King which he has requested me to read:—"As you are presiding at a meeting of the Prince of Wales's London Hospital Fund pray express to the Council the unabated interest I take in its continued success and prosperity." I am also asked to announce the fact that the Duke of Cornwall and York has kindly consented to act in the future as the President of the Fund, and I have no doubt His Royal Highness will, if possible, preside at the future meetings.

Lord ROTHSCHILD, in moving a resolution of thanks to His Majesty the King, said: My Lords and gentlemen, I have great pleasure in rising to propose a resolution which I am sure you will all agree to unanimously, namely, to express to His Majesty the King our thanks for his gracious telegram and for the continued interest he takes in this Fund. Rising as I do I should be failing in my duty if I did not ask His Grace the Duke of Fife to express to the King the thanks of the Council, the thanks of the hospitals, and the thanks of the patients for having started this Fund, and for the great and continued interest he has always taken in it. (Hear, hear.) If the Fund did not reach those proportions which the King wished it to do there were circumstances which prevented it. Great claims on the generosity of the public have arisen since the Fund was started which has perhaps prevented it reaching those dimensions which the King wished. When the Fund was started the great and generous idea of our Sovereign was that a sufficient sum should be collected, chiefly by the means of annual subscriptions, to place all hospitals beyond want. In addition to that generous idea the King's noble thought was that those hospitals should be helped which do the most for the suffering patients, and that the Council of this Fund should be instrumental in improving the position of the hospitals. I think I may say without fear of contradiction that the funds placed at the disposal of the Council have been given to those hospitals which do most and which do the best for the suffering poor. I think the work of the visitors has considerably improved the state of the hospitals. Having said these few words I call upon you, my lords and gentlemen, to adopt unanimously the vote of thanks to our gracious Sovereign for the kind and continued interest he takes in this great undertaking.

Mr. HUGH COLIN SMITH seconded the resolution.

The HONORARY SECRETARY (Mr. J. G. Craggs) having read the minutes of the last meeting, upon the motion of the Chairman,

Mr. H. C. SMITH read the report of the General Council for the year ending 1900 as follows:—

Since the end of 1900, the fourth year of this Fund, the General Council have occasion to mourn the loss of our beloved Sovereign, her Gracious Majesty Queen Victoria, who was the first Patron of this Fund, and in whose honour it was founded as a means of commemorating the Sixtieth Year of her Reign.

The words used on February 5th, 1897, by the Royal Founder, our present Sovereign, may be quoted:—

"Having ascertained from the Queen that she has no wish to express a preference for any one of the many proposals loyally suggested for commemorating, nationally or locally, the sixtieth year of her reign, I feel at liberty to bring to the notice of the inhabitants of the metropolis a project lying very near my heart, its object being to attach the sentiment of gratitude for the blessings which the country has enjoyed during the last sixty years to a scheme of permanent beneficence."

The General Council hope that year by year the Fund will continue to surely and steadily grow and become a permanent memorial of the blessings of the long and beneficent reign of our late Sovereign, and one worthy of the great progress made by the nation during the years she reigned over it.

The war in South Africa has been continued throughout the year, and the public have been asked for considerable sums in connection with the various funds originating in this cause.

For this and other reasons it is very satisfactory to be able to record an increase of £3,000 in the total receipts for the year. Excluding the initial start in the Jubilee Year, the figures show continuous progress, viz.:—1898, £39,272; 1899, £48,536; 1900, £51,549.

Although the receipts were only £3,000 in excess of last year, it was felt that the claims of the hospitals justified a larger distribution than before, and it was decided to award to the hospitals and convalescent homes £50,000, as compared with £42,000 last year, being an increase of £8,000.

No appeal was made to the public for eighteen months, so as to avoid any clashing with the various war funds, but it was felt at the end of that time, shortly before the close of the year, that it would not be unfair to make an appeal, and one was issued on November 22nd. The response to this was somewhat disappointing, but with some special gifts made shortly before the end of the year, it was only necessary to withdraw from the balance of the funds in hand last year a sum of £2,183 8s. 4d. in order to make up the distribution of £50,000.

The League of Mercy was enabled to contribute £6,000 this year, as compared with £1,000 in the previous year.

The grants made to the various hospitals appear in the Report of the Hospitals Distribution Committee, and it will be seen that the additional sum of £8,000 placed at the disposal of the committee has been distributed this year mainly in the way of donations, because the special circumstances of the year have tended to lessen the support given by the public to individual hospitals. At the same time the grants are in many cases made with a view to the improvement of the various institutions, as well as providing for the maintenance of the 287 additional beds which have been re-opened with the help of this Fund since its inception.

This Fund has continued its sympathy to the movement in favour of the abolition of the heavy rating of hospitals.

The expenditure for the year has been £1,960, as compared with £1,853, or about £100 more. It must be remembered that last year's expenditure was considered a minimum. In comparing the ratio of expenditure to the sums received the result is practically identical, viz., 3⅝ per cent. in each case.

In considering the expenditure there must be borne in mind not only the work required to collect and record the subscriptions and donations, the necessity of a certain amount of advertising for contributions and for announcing the sums received, but also the heavy statistical and other work that has to be done in connection with the annual inspection of the hospitals applying for grants and the consideration of their respective needs and merits. The expenditure will compare favourably with that of other institutions.

In addition to the statement of the annual accounts there has been added this year comparative tables of receipts and payments for the four years during which the Fund has been established, and also particulars with regard to legacies that have been announced, both of which it is thought will prove interesting.

The Council wish to express their thanks to the following gentlemen, who have given their services as hospital visitors during the year 1900:—Lord Lister, Sir William MacCormac, Bart., P.R.C.S., Sir Thomas Smith, Bart., Sir W. S. Church, M.D., P.R.C.P., John Croft, F.R.C.S., A. B. Duffin, M.D., Christopher Heath, F.R.C.S., N. C. Macnamara, F.R.C.S., Dr. Pavy, F.R.S., Dr. E. C. Perry, T. Pickering Pick, F.R.C.S., H. Power, F.R.C.S., Alfred Willett, F.R.C.S., Viscount Duncannon, C.B., Lord Sandhurst, G.C.I.E., Sir Trevor Lawrence, Bart., Sir Henry Burdett, K.C.B., Thomas Burt, M.P., Charles Burt, J. G. Craggs, F.C.A., Frederick M. Fry, W. Douro Hoare, H. J. King, James C. Marshall, J. Pierpont Morgan, jun., Albert G. Sandeman, Hugh C. Smith.

Some hope is felt that this fund, which is chiefly one supported by annual subscriptions, may be able to show increased revenue from this source each year, as the objects of the fund become better understood and appreciated. One of these objects is, while avoiding the diversion of individual interest from particular charities, to secure by personal and impartial inspection that moneys are only awarded to institutions that properly deserve encouragement and support.

In this connection it was pleasant to receive a letter from the Worshipful Company of Drapers enclosing a cheque for £1,000 for an annual subscription at the pleasure of the Court of Assistants, accompanied with a letter in which it was stated:—

"The Company learnt, with much satisfaction, from the last Report of the Prince of Wales's Hospital Fund, that the committee have appointed visiting sub-committees, consisting of persons practically acquainted with hospital management, with the object of obtaining information as to the merits and needs of the various institutions. The Company regard this as a most important step towards promoting the well-being of Metropolitan Medical Charities and strengthening their claims on public benevolence."

While the work of this Fund is limited to the London hospitals, yet the hospitals themselves are a centre of very wide influence, extending far beyond the limits of London; indeed, to a greater or less extent they affect the well-being of the whole Empire, as they supply no inconsiderable proportion of the medical men whose services are afterwards devoted to all parts of the Empire.

In conclusion, the Council desire to record again the kind gratuitous assistance they have had from the Press in carrying on the work of the Fund. Some of the Press comments upon the work of the preceding year are included at the end of the information accompanying this report.

Lord Farquhar in seconding the adoption of the report said: I hope, and we all hope and trust that the war which has engaged so much attention, and has naturally drawn such large subscriptions from all private people for nearly two years is now coming to an end, and therefore more funds will be available for this very excellent cause that we are all here to support to-day. I must say that part of the report read by Mr. Smith appeals to me very much indeed, namely, the letter from the Worshipful Company of Drapers expressing their great approval of the way in which the hospitals have been visited, and the manner in which the Committee have awarded the different sums. I think that is a very important part of the report. I hear it has given satisfaction all over London, and that being the case, I feel how much indebted we are to the gentlemen who visit the hospitals and who manage this part of the business.

The Rev. T. Bowman Stephenson, Sir Trevor Lawrence Sir Henry Burdett and Mr. J. G. Craggs having spoken on the motion, it was put to the meeting and carried unanimously.

On the motion of Lord Rothschild, seconded by Lord Reay, the accounts and balance-sheet were adopted and passed.

Lord Duncannon moved that Mr. A. D. Fripp, F.R.C.S., and Sir Savile Crossley (both of whom had returned from South Africa) be added to the list of visitors, and said that His Royal Highness the Duke of Cornwall and York had gladly consented to become President of the Fund and had expressed the hope that the gentlemen who had so kindly acted last year would continue to undertake this most important duty.

Lord Iveagh having seconded the motion,

The Chairman said: I beg now to convey H.R.H. the President's best thanks to the General Council, the honorary treasurer, the honorary secretaries, and honorary officials for their services during the past year on the termination of the year of office, and to invite them to serve again. May I ask those gentlemen to whom this motion refers not to measure the appreciation in which their services are held by the brevity of my remarks, but as I am acting to-day entirely for another I feel it is only right I should limit myself strictly to the execution of the commission with which I have been entrusted. I beg to convey to those gentlemen mentioned the Duke of Cornwall and York's best thanks for their services.

Lord Rothschild said, on behalf of his colleagues and himself he thanked His Grace the Duke of Fife for the kind words he had conveyed to them from His Royal Highness the Duke of Cornwall and York. He felt sure that His Royal Highness, following the footsteps of His Majesty the King, would ensure to the Council a long and prosperous career.

Mr. Sydney Buxton, M.P., proposed a vote of thanks to His Grace for presiding, which was seconded by Sir John Aird.

The Chairman, in reply, said: I beg to thank you sincerely for the kind manner in which you have received the vote of thanks proposed by Mr. Sydney Buxton and seconded by Sir John Aird. As I only joined the council last year as an *ex officio* member in my capacity as Lord Lieutenant of London I have only attended one meeting, and therefore I am not as conversant as I should wish with the general work of the Fund. Under those circumstances, and for other reasons, I will not occupy your time any further, but I will merely express my best wishes for the continued prosperity of the good work of this Fund. (Hear, hear.)

The proceedings then terminated.

